# 

**Published:** 1877-08-20

**Authors:** 


ORIGINAL AND SELECTED ARTICLES—

Progressive Pharmacy (sugar-coated pills of the day). By Wm. A. Greene, M. D., Macon, Ga .... 197
Fluid Extract Kidney Leaf Comp. By Wm. A. Greene, M.D., Macon, Ga .... 199
Review of Current Neurological Matters. Condensed by C. C. Vanderbeck, M.D .... 201
Materialistic Psychology. By A. Donald, M.D., Opelousas, La .... 203
Case of Typhoid Fever. By Z. D. Herndon, M.D., Richmond, Va .... 204
Treatment of Carbuncle. By C. B. Leitner, M.D., Columbus, Ga .... 205
Necessity of Caution in the Employment of Chloroform During Labor. By. W. T. Lusk, M.D., New York .... 206
Gelseminum. By Dr. Adolphus .... 207
Note on the Automatic Reduction of Hip-Joint Luxation. By H. H. A. Beach, M. D. .. 208

ABSTRACTS AND GLEANINGS—

Craniotomy, 209. A Sign of Early Pregnancy, 210. Enteric Fever—Is It Contagious?; Jaborandi, 212. Bromide of Ethyl as an Anæsthetic; Santonin, 214. Trepanning for Fracture of the Skull; Treatment of Croup by Eucalyptus, 215. Chloral in Convulsions; Properties of the Human Gastric Juice; Profuse Sweating, 216. Cocoa as a Food for Infants; Strictures; A New Method of Treating Nasal Catarrh; Ecbolic Action of Quinia, 217. Action of Butylchloral; Chronic Intermittents; Influence of Artificial Suppression of the Cutaneous Secretion; Scrofulous Ulcers—Red Lead and Cinnabar Plaster, 218. Inebriety, 219. Chloral in Eclampsia. Something New About Oxygen, 220. Choice of Sedatives for the Very Young.

PRACTICAL NOTES AND FORMULÆ—

Thompson's Eye Water; Toothache Drops; Infantile Colic; Tonic Doses of Mercury 221. A Useful Paste; Diet for Infants; Sedative Antiseptic; Flaxseed Tea; Cold Milk Punch; Urates; Oxalate of Lime; Urate of Lime, 222. Slippery Elm Jelly and Tea; Wine Whey; Carb. Ammonia in Snake Bite; Disinfectant for Privies; Nitric Acid Test for Albumen; Duration of Pregnancy; For Ringworm; For Haematuria; Urinary Deposits; Diptheria; Brown Discoloration of Skin; Sebaceous Glands; Bromide of Iron; For Acne, 223. Chronic Diarrhœa; Catarrh Snuff; Carboli[illegible] Collodin; Sore Nipples; Toothache Drops; Salivation; Urate of Ammonia; Obstinate Vomiting, 224.

SCIENTIFIC ITEMS—

Man's Average Height; A New Marine Distress Signal; Temperature of the Interior of the Earth; Davyum, 224.

EDITORIAL AND MISCELLANEOUS—

Special Notice To Our Editorial Brethren—Postal Annoyances; Water; Medical Societies; To the Country Practitioner, 225. Medical Colleges; A Letter; Monobrominated Camphor in Summer Complaints of Children; Potentilla Canadensis; Albuminous Conditions of the Blood, etc.; Inebriate Asylum; Book Notices and Pamphlets Received, 226.

